# Suicidal *Leishmania*

**DOI:** 10.3390/pathogens9020079

**Published:** 2020-01-25

**Authors:** Lucie Podešvová, Tereza Leštinová, Eva Horáková, Julius Lukeš, Petr Volf, Vyacheslav Yurchenko

**Affiliations:** 1 Life Science Research Centre and Institute of Environmental Technologies, Faculty of Science, University of Ostrava, 710 00 Ostrava, Czech Republic; 2 Department of Parasitology, Faculty of Science, Charles University, 128 44 Prague, Czech Republic; 3 Biology Centre, Institute of Parasitology, Czech Academy of Sciences, 370 05 České Budějovice (Budweis), Czech Republic; 4 Faculty of Sciences, University of South Bohemia, 370 05 České Budějovice (Budweis), Czech Republic; 5 Martsinovsky Institute of Medical Parasitology, Tropical and Vector Borne Diseases, Sechenov University, 119435 Moscow, Russia

**Keywords:** *Leishmania mexicana*, suicidal system, ecDHFR, BnSP-7, apoptosis

## Abstract

*Leishmania* are obligate intracellular parasites known to have developed successful ways of efficient immunity evasion. Because of this, leishmaniasis, a disease caused by these flagellated protists, is ranked as one of the most serious tropical infections worldwide. Neither prophylactic medication, nor vaccination has been developed thus far, even though the infection has usually led to strong and long-lasting immunity. In this paper, we describe a “suicidal” system established in *Leishmania mexicana*, a human pathogen causing cutaneous leishmaniasis. This system is based on the expression and (de)stabilization of a basic phospholipase A2 toxin from the *Bothrops pauloensis* snake venom, which leads to the inducible cell death of the parasites in vitro. Furthermore, the suicidal strain was highly attenuated during macrophage infection, regardless of the toxin stabilization. Such a deliberately weakened parasite could be used to vaccinate the host, as its viability is regulated by the toxin stabilization, causing a profoundly reduced pathogenesis.

## 1. Introduction

Leishmaniasis is a neglected tropical disease, caused by *Leishmania* spp., flagellated protozoan parasites belonging to the family Trypanosomatidae [1,2]. The transmission occurs through the bite of a female sand fly (Diptera: Phlebotominae). In the vector, flagellated promastigotes differentiate into the virulent metacyclic forms, which upon injection into the mammalian host develop into the aflagellated, non-motile amastigotes that reside in the phagolysosomes of host macrophages [3,4]. The extent of clinical manifestations of the disease depends both on the parasite species and the immune response developed by the host, but typically ranges from the self-healing cutaneous lesions to fatal visceral infections [1]. Nowadays, leishmaniasis represents the second most serious tropical parasitic disease in terms of mortality and, according to the World Health Organization, over 350 million people live in at-risk endemic areas [5]. Current treatment of the disease is expensive and often associated with severe adverse side effects [6]. Moreover, in many instances a long-lasting parasite resistance develops, underlying the need for an efficient vaccination approach that would allow proper control and prevention of this debilitating disease [7].

To date, several anti-*Leishmania* vaccination strategies have been proposed. Leishmanization, which is based on the inoculation of low-titer or attenuated live virulent parasites, represents the most efficient approach [8,9]. However, its applicability is limited due to apparent safety concerns [10]. Vaccination trials based on killed or heat-inactivated parasites, recombinant proteins, and DNA vaccines have shown limitations in murine models and field studies [11]. Another promising experimental approach to this problem is a usage of the genetically modified *Leishmania* lines as attenuated vaccines [12,13,14,15,16]. There are two aspects that make generation of a successful vaccine particularly challenging. On the one hand, live virulent *Leishmania* cells are needed to trigger and facilitate complete host immune response, while, on the other hand, such infection must be controllable, so that it does not result in the development of persistent lesions and/or immunosuppression. An inducible expression of toxic genes from suicidal cassettes and subsequent production of suicidal *Leishmania* strains have been suggested to provide an ideal solution for the aforementioned vaccination limitations: the parasites possess a complete repertoire of virulent components, yet, once the host is immunized, they can be inducibly killed via the intrinsic apoptotic pathways [17,18,19,20].

In this study, we introduce an inducible suicidal system in *Leishmania mexicana*, a causative agent of cutaneous leishmaniasis. A similar system has recently been established in another trypanosomatid species, *Trypanosoma cruzi* [21]. In both systems, a destabilization domain, derived from the *Escherichia coli* dihydrofolate reductase (ecDHFR) destabilizes the fusion protein and facilitates its degradation by proteasomes. This process can be reversed by the addition of a specific ligand, Trimethoprim (TMP), or its derivate, Trimethoprim-lactate (TMP-lac) [22]. Screening of a panel of toxins and antimicrobial peptides (AMPs) under axenic conditions revealed that one of them, a basic phospholipase A2 (PLA_2_) from the *Bothrops pauloensis* snake venom, called BnSP-7, caused apoptosis-like cell death of the parasites upon the addition of the stabilizing ligand. Noticeably, this toxin has previously been demonstrated to kill *L. amazonensis* promastigotes in vitro, as well as to delay the amastigote-to-promastigote differentiation, to induce ultrastructural changes in promastigote morphology, and to reduce virulence [23]. The data presented here are complementary to the previous findings. In macrophage infections, the BnSP-7 expressing mutants of *L. mexicana* showed a significantly reduced infectivity rate compared to its wild type counterparts, even in the absence of the stabilizing agent. Importantly, this attenuated phenotype was further reinforced by the addition of TMP-lac.

We believe that the suicidal system presented here and the resultant of strongly attenuated parasites can be used to develop a novel vaccination strategy against *L. mexicana,* and provide additional insights into the host-derived immune response after elimination of the pathogen. We acknowledge that our findings must be properly reexamined in vivo in animal models of leishmaniasis. 

## 2. Results

### 2.1. Not All Toxins/Antimicrobial Peptides Are Toxic for *Leishmania mexicana* Promastigotes

Growth kinetics was measured for five transgenic *L. mexicana* cell lines, expressing α-toxin, Cecropin-A, Attacin-A, BnSP-7, and Cathelicidin-5, in the presence or absence of the toxin stabilizers, TMP or TMP-lac. We were not able to establish a *Leishmania* cell line expressing Exotoxin-A, even after multiple attempts. This was probably due to insufficient destabilization and, thus, background expression of the toxin, which resulted in immediate parasite death upon transfection. Of note, all other toxins, fused to ecDHFR-HA, are relatively small compared to the Exotoxin-A, implying that degradation of the toxin-destabilizing domain fusion may be influenced, inter alia, by the toxin size. The growth curves demonstrated that upon addition of the inducer all but one (BnSP-7) toxin-expressing cell lines divided similarly to the wild type *Leishmania* (Figure S1). The cell morphology was also not affected, supporting the notion that the inducer itself is not detrimental to the parasites. We concluded that α-toxin, Cecropin-A, Attacin-A, and Cathelicidin-5 were not toxic for *L. mexicana* promastigotes. 

### 2.2. BnSP-7, a Basic Phospholipase A2 from *Bothrops pauloensis* Venom, Dramatically Reduces Viability of *L. mexicana* Promastigotes *In Vitro*

**Figure 1:**
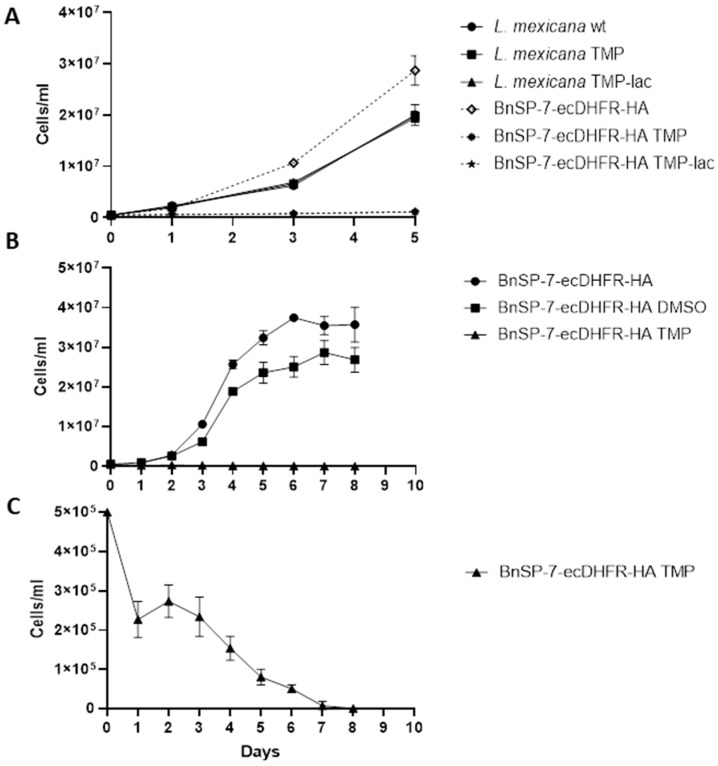
Growth kinetics of *Leishmania mexicana* expressing the BnSP-7 toxin. (**A**) Growth curves of the wild type and BnSP-7-ecDHFR-HA-expressing *L. mexicana* after induction with 20 μM Trimethoprim (TMP)/Trimethoprim-lactate (TMP-lac); (**B**) growth curves of BnSP-7-ecDHFR-HA-expressing *L. mexicana*, cultivated for 8 days with continuous replenishment of TMP or DMSO; (**C**) TMP facilitates decrease in a number of BnSP-7-ecDHFR-HA-expressing *L. mexicana*. All data are derived from three independent biological replicates. The error bars indicate standard deviations.

The growth of the BnSP-7-ecDHFR-HA-expressing cells was significantly inhibited upon the addition of either TMP or TMP-lac, as compared to the wild type *L. mexicana* or the untreated promastigotes (Figure 1A,B; Table S1). In the absence of induction, the BnSP-7-ecDHFR-HA-expressing cells grow better than the parental ones. When performing an extended growth kinetics analysis, we noticed that the starting concentration of parasites (5 × 10^5^ cells/mL) was decreasing even after 24 hours of TMP treatment (Figure 1C), with no viable cells observed on day 8, whereas the untreated group had reached the stationary log phase on day 6 (Figure 1C). We conclude that BnSP-7 toxin is killing *L. mexicana* promastigotes *in vitro*. 

**Figure 2:**
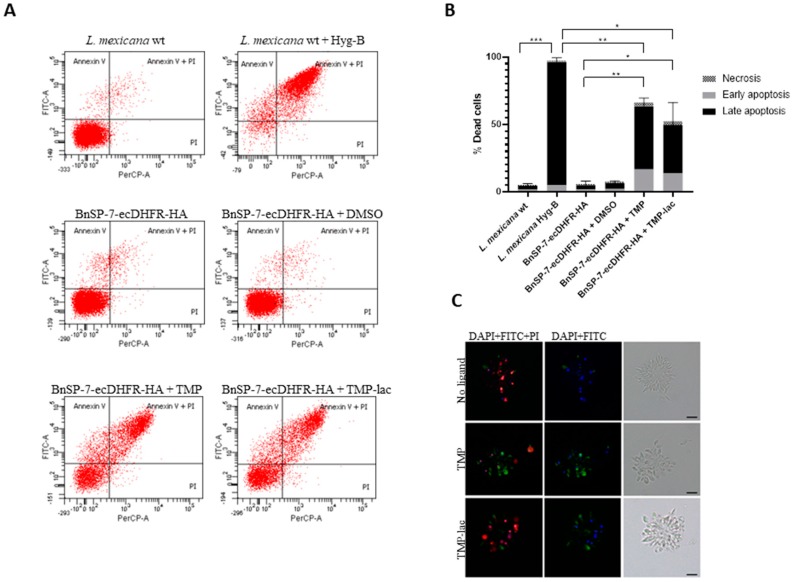
Cell death of *L. mexicana* expressing the BnSP-7 toxin. (**A**) Flow cytometry analysis of the *L. mexicana* cells treated with TMP or TMP-lac for 5 days. Hygromycin B treated wild type *L. mexicana* cells were used as an apoptotic/necrotic control; (**B**) apoptosis and necrosis were quantified after FITC-Annexin V and PI labeling; bar graphs represent mean ± SD in three independent experiments; asterisks indicate *p* values; * ≤0.05, ** ≤0.01, *** ≤0.001; (**C**) fluorescence microscopy of the Annexin V-FITC, PI, and DAPI (left panels), Annexin V-FITC and DAPI (middle panels) stained, and light microscopy controls (right panels) of the *L. mexicana* BnSP-7-ecDHFR-HA cells prior and after induction by the stabilization ligand for 48 hours. Scale bars are 10 μm.

To confirm this, we checked the early as well as the late apoptotic events by fluorescence microscopy and flow cytometry using the FITC-Annexin V/PI method [24] (Figure 2). As a positive control, the wild type *L. mexicana* treated with Hygromycin B was used. Cells undergoing apoptosis became abundant in both TMP and TMP-lac-treated conditions. We also noticed that TMP-lac showed slightly lower efficacy, compared to TMP (Figure 2). However, we continued working with TMP-lac due to its solubility in water and, thus, direct applicability for downstream in vivo experiments. 

### 2.3. BnSP-7-ecDHFR-HA System Is Functional in Axenically Differentiated *L. mexicana* Amastigotes

**Figure 3:**
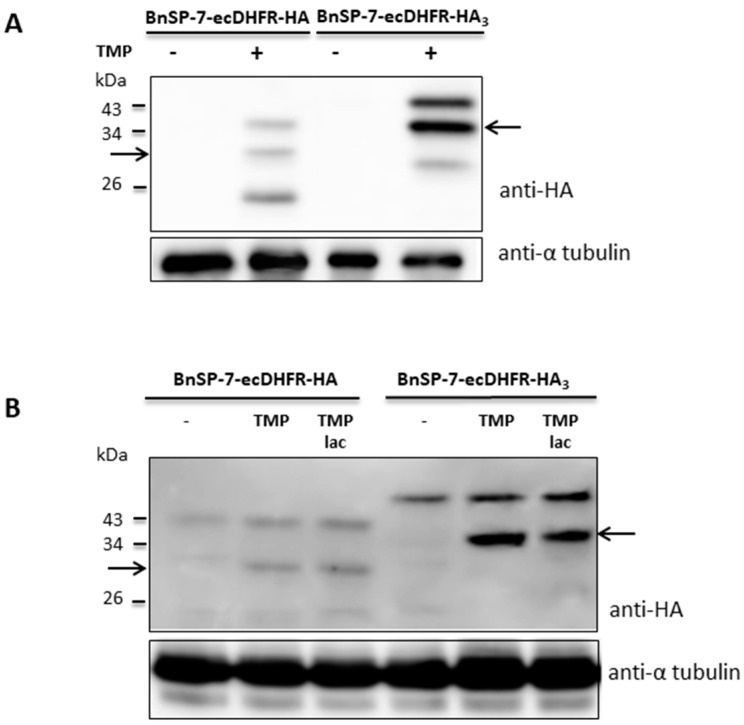
Protein levels of BnSP-7-ecDHFR-HA and BnSP-7-ecDHFR-HA_3_ after induction by TMP/TMP-lac in *L. mexicana.* (**A**) Western blot of the untreated promastigote lysates and those treated with 20 μM TMP for 48 hours; (**B**) Western blot of the untreated amastigote lysates and those treated with 20 μM TMP for 48 hours. Tubulin served as a loading control. Arrows indicate the band of the expected size (32 kDa and 34 kDa for HA- or HA_3_-tagged proteins, respectively).

To check whether stabilization of the toxin protein was not restricted to the promastigote stage, the BnSP-7-ecDHFR-HA-expressing *L. mexicana* cells were differentiated into amastigotes *in vitro* and, subsequently, treated with TMP/TMP-lac for 72 hours. Differentiation from the promastigotes into amastigotes in the presence of TMP/TMP-lac was not possible, since the cells died within several days of treatment (Figure 1 and Figure 2). Functionality of the stabilization system was checked by Western blotting, detecting properly processed BnSP-7-HA or BnSp-7-HA_3_ in both promastigotes (Figure 3A) and cells that were in vitro differentiated into the amastigotes upon the treatment with either TMP or TMP-lac (Figure 3B). The triple HA-tag was introduced for more efficient pull-down. In all cases, protein bands of the expected size, namely ~32 kD for BnSP-7-HA and ~34 kD for BnSp-7-HA_3_, were documented after the TMP/TMP-lac induction. Moreover, the anti-HA antibody also detected a higher (~40 kD) and a lower molecular weight band (~20 kD) in the TMP/TMP-lac-treated parasites. Mass spectrometry analysis showed that these bands represent ubiquitylated and partially degraded forms of BnSP-7-HA or BnSP-7-HA_3_. 

### 2.4. The BnSP-7-ecDHFR-HA-expressing *L. mexicana* Are Attenuated in Macrophage Infections

**Figure 4:**
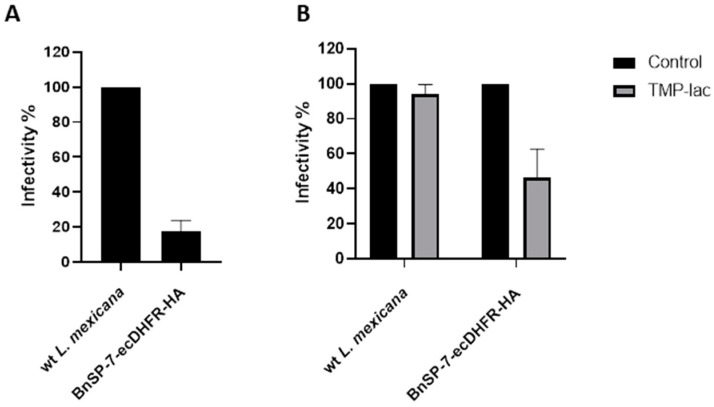
Relative rates of *L. mexicana* infectivity. (**A**) Macrophage infection by the wild type and the BnSP-7-ecDHFR-HA-expressing cells; (**B**) macrophage infection by the wild type and the BnSP-7-ecDHFR-HA-expressing cells treated with 20 μM TMP-lac for 48 hours. Data are presented as percentage of the wild type parasites (A) or saline-treated controls (B), taken at 100%. The error bars indicate standard deviation derived from the two independent biological experiments (with 3 technical replicated each).

Next, J774 macrophages were infected with either the wild type or the BnSP-7-ecDHFR-HA-expressing *L. mexicana*, followed by the treatment 24 hours post infection (p.i.) with either 20 μM TMP-lac or saline buffer, as a control. Four hours p.i., the cells were checked for the proper phagocytic activity and no parasites were observed outside of the macrophages. Importantly, the total number of counted amastigotes (a proxy of *Leishmania* infectivity) 72 hours p.i. was substantially lower in the case of *L. mexicana* BnSP-7-ecDHFR-HA as compared to the wild type parasites even without the TMP or TMP-lac treatment (Figure 4A). This implies higher susceptibility of the mutant cells towards macrophage phagocytosis machinery and may reflect the incomplete toxin destabilization inside the macrophages, as the percentage of infected macrophages within the examined groups did not differ (Figure S2). The two-day cultivation of the infected macrophages with 20 μM TMP-lac resulted in a ~60% reduction of the recovered *L. mexicana* amastigotes, when the number of the BnSP-7-ecDHFR-HA-expressing parasites was compared to their wild type kin (Figure 4B).

## 3. Discussion

Toxins and AMPs are natural substances of both prokaryotic and eukaryotic origin [25,26]. Their role is generally related to offensive/defensive responses, resulting in damage or death of the targeted organism [27,28]. Some toxins or AMPs are able to kill trypanosomatid parasites, and their trypanocidal and/or leishmanicidal properties have been extensively studied [29]. However, these studies were mostly restricted to the in vitro killing assays with recombinant or synthetic proteins, limiting them primarily to the therapeutic applications. 

The aim of this study was to express different toxins/AMPs in *L. mexicana* using a recently established inducible protein stabilization system [22]. Such genetically-modified suicidal strain of *Leishmania* would be an eligible and promising candidate for vaccine development. Our search for proteins, whose killing activity was reported either for *Trypanosoma* or *Leishmania* spp., resulted in a list of six potential candidates: α-toxin, Attacin-A, BnSP-7, Cecropin-A, Cathelicidin-5, and Exotoxin-A. Of note, three of these toxins (α-toxin, Cecropin-A, and Exotoxin-A) have been previously tested in *T. cruzi* [21].

Insect AMPs Attacin-A, isolated from *Glossina morsitans morsitans*, and Cecropin-A, obtained from *Hyalophora cecropia*, fused to the *E. coli* DHFR degron, did not change the viability of *Leishmania mexicana* promastigotes upon protein stabilization (Figure S1). They have been previously demonstrated to inhibit growth of *T. brucei* and *T. cruzi* [30,31], as well as *L. panamensis* and *L. major* amastigotes [32] in vitro. A *Bos taurus-*derived antimicrobial peptide Cathelicidin-5, whose lethal activity was previously demonstrated for *L. major* promastigotes [33], was similarly not effective in *L. mexicana* (Figure S1). AMPs are mostly cationic agents disrupting the membrane integrity by the electrostatic interactions with their anionic phospholipid counterparts [34,35]. Different membrane composition in the *Leishmania* and *Trypanosoma* cells (or in different *Leishmania* spp.) may be responsible for the discrepancy observed for these parasites. This implies that there must be other mechanisms (for example, post-translational modifications, protein stability and folding, etc.), determining whether a toxin will be active against a given parasite species. To illustrate this further, the α-toxin from *Staphylococcus aureus* was the best-choice candidate in *T. cruzi* studies [21], whereas it had no effect on *L. mexicana* (Figure S1). This toxin is known to form large Ca^2+^-permissive pores in the cellular membrane, resulting in a massive homeostasis disruption and cell death [36]. Because the α-toxin forms a heptameric transmembrane pore [37], we presumed that its fusion with a destabilizing domain has interfered with its proper folding. In contrast, all our attempts to generate a cell line, expressing *Pseudomonas aeruginosa* Exotoxin-A, have invariably failed. We explained this by insufficient toxin destabilization, followed by the inhibition of protein synthesis and cell death. Overall, the data presented here indicate that the functionality of a suicidal system relies both on the selected toxin and the host used for its expression. 

The BnSP-7, a catalytically inactive Phospholipase A2 (PLA_2_) homolog from the *Bothrops pauloensis* snake venom, was the only, albeit very effective, toxin, acting against *L. mexicana* promastigotes and amastigotes, identified in this study. Although lacking the catalytic activity due to a single amino acid substitution (Asp49Lys), myotoxic PLA2 homologs have the ability to disrupt biological membranes in a Ca^2+^-independent manner not involving the hydrolysis of phospholipids [38,39,40]. In cytotoxicity assays, its activity has also been demonstrated against several *Leishmania* spp. [41,42,43]. The pilot TMP/TMP-lac treatment experiments with the BnSP-7-ecDHFR-HA-expressing parasites have confirmed the anti-leishmanial potential of this toxin (Figure 1). Cells did not multiply and their morphology dramatically changed from long promastigotes to round-shaped cells, indicating apoptosis. Moreover, the number of parasites was decreasing after 72 hours of continuous TMP treatment, reaching an undetectable level by day 8 (Figure 1C). Flow cytometry and fluorescence microscopy evaluation [44,45] confirmed the apoptotic nature of the toxin-induced *Leishmania* cell death (Figure 2). 

Amastigotes could not be differentiated in the presence of BnSP-7. To overcome this, we first produced amastigotes in vitro and then treated them with either TMP or TMP-lac. The readout of this experiment was a stabilized toxin, detected by immunoblotting. Both the promastigotes and the amastigotes showed the same expression pattern with three bands upon addition of the stabilizing agent (Figure 3). Immunoprecipitation followed by mass spectrometry analysis confirmed the identity of BnSP-7-ecDHFR-HA, its degradation product, and the post-translationally modified mono-ubiquitylated form. In both promastigotes and amastigotes, the functional toxin was predominant after stabilization. Recent studies have shown that mono-ubiquitylation of smaller proteins can target them for proteasomal recognition and processing [46]. We assume that mono-ubiquitylation of the fused destabilized protein drives its proteolysis, and the domain stabilization by TMP/TMP-lac interferes with this process. 

When performing the macrophage infections, we noticed a surprisingly reduced infectivity of the BnSP-7-ecDHFR-HA-expressing *L. mexicana* even in the absence of the TMP-facilitated stabilization (Figure 4A). Since the phagocytic activity of macrophages did not differ between the wild type *L. mexicana* and the mutant, we presume that the BnSP-7-ecDHFR-HA-expressing parasites possess all antigens required for macrophage recognition, but their viability and/or division rate was lowered after internalization into the phagolysosomes. The most parsimonious explanation of such an attenuated phenotype is that the toxin was partly stabilized in the phagolysosomal environment, thus resulting in a reduced infectivity. As expected, the TMP-lac treatment further decreased the number of recovered amastigotes in the case of the mutant cell line compared to the wild type *L. mexicana* (Figure 4B). The BnSP-7 toxin expression causes elevated susceptibility upon macrophage engulfment and this can be enhanced via an inducible (de)stabilization system. We realize that such a highly attenuated mutant strain of *L. mexicana* needs to be reexamined in vivo in animal models of leishmaniasis, as the immune response is too complex to be reliably predicted from the in vitro studies only.

In conclusion, this is the first report of the basic PLA_2_ homologue, derived from the *Bothrops pauloensis* snake venom, with lethal activity against *L. mexicana*. We believe that these results may ultimately lead to the development of novel vaccination strategies based on the controllably suicidal *Leishmania*. 

## 4. Materials and Methods 

### 4.1. Axenic Cultivation of *Leishmania mexicana*

*Leishmania mexicana* (MNYC/BZ/1962/M379) promastigotes were cultured at 23 °C in the M199 medium (Sigma-Aldrich, Saint. Louis, MO, USA), supplemented with 2 µg/mL Biopterin (Sigma-Aldrich), 2 µg/mL Hemin (Jena Bioscience GmbH, Jena, Germany), 25 mM HEPES, 50 units/mL of penicillin/streptomycin (Life Technologies, Carlsbad, CA, USA), and 10% Fetal Bovine Serum (FBS, BioSera Europe, Nuaillé, France). The amastigotes of *L. mexicana* were differentiated in vitro and cultivated at 32 °C in the complete M199 medium (pH 5.5), supplemented with 20% FBS [47,48]. The following ligands were tested: Trimethoprim (Sigma-Aldrich, 10 mM stock in DMSO) and Trimethoprim-lactate (TMP-lac, Sigma-Aldrich, 10 mM stock in M199, or 1 mM stock in a saline buffer).

### 4.2. Genetic Manipulations

Information on the toxins and AMPs analyzed in this study is summarized in Table 1. The open reading frames (ORFs) of α-toxin, Cecropin-A, and Exotoxin-A were PCR amplified from the pTREX-based constructs (kindly provided by Dr. Huang [21]) using primers A1/A2, C1/C2, and E1/E2, respectively (Table S2). These fragments were fused with the modified ecDHFR-HA domain (mut2 in [22]), amplified from the pLEXSY-based construct with primers D1/D2, using nested primers A3, C3, E3, and D3 (Table S2) as described previously [49]. The fragments encoding ecDHFR-HA (mut2) fusions of Attacin-A, BnSP-7, and Cathelicidin-5 (GenBank accession numbers AY607104, AF145781, and X97609, respectively), were synthesized de novo by GeneCust Europe (Boynes, France). All fragments were cloned into the pLEXSY-hyg2 (Jena Bioscience GmbH) using restriction enzymes *Bgl*II (Thermo Fisher Scientific, Waltham, MA, USA) and *Not*I (New England Biolabs, Ipswich, MA, USA). All resulting plasmids were linearized with *Swa*I (New England Biolabs), gel purified, and transfected into the log phase-grown procyclic promastigotes of *L. mexicana* using BTX ECM 630 electroporator (Harvard Apparatus Inc, Holliston, MA, USA), as described previously [50]. The transfectants were selected for 10 days in the cultivation medium, supplemented with 200 μg/mL of Hygromycin B (Carl Roth GmbH, Karlsruhe, Germany). 

In addition, the BnSP-7-ecDHFR-HA fragment was re-amplified from the pLEXSY construct using primers B1 and D4. The resulting HA_3_-tagged construct was cloned back into the pLEXSY-hyg2 plasmid and transfected as described above. PCR confirmation of the correct integration was performed using primers SSU_F and HA_R (Table S2).

### 4.3. Growth Kinetics and Differentiation 

Growth kinetics analysis of the wild type and transgenic cell lines was performed for 5 days from the starting density of 5 × 10^5^ cells/mL in three biological replicates, as described previously [51]. The counting was also performed for all transfectants after the addition of 20 μM TMP or TMP-lac on day 0, using wild type *L. mexicana* as a control. The BnSP-7-ecDHFR-HA expressing *L. mexicana* cells were additionally counted in the same manner every 24 hours for 8 days with daily replenishment of TMP. 

Differentiation of the BnSP-7-ecDHFR-HA expressing *L. mexicana* mutants into amastigotes was performed by varying pH and temperature as described previously [48]. Once the parasites reached the amastigote stage (day 17), they were treated with 20 μM TMP/TMP-lac and analyzed by Western blotting after 48 hours.

### 4.4. Apoptosis Assays

The analysis of *L. mexicana* apoptosis was carried out using the eBioscience Annexin V Apoptosis Detection Kit FITC (Thermo Fisher Scientific) according to the manufacturer’s instructions. Briefly, 5 × 10^6^/mL of the BnSP-7-ecDHFR-HA expressing promastigotes, incubated with or without 20 μM TMP, TMP-lac, or DMSO, were harvested, washed, and treated with 5 μL of FITC-Annexin V for 15 min at room temperature in the dark, followed by treatment with 5 μL propidium iodide (PI, 20 µg/mL). The cells were then analyzed on a FACSCanto II flow cytometer (Becton Dickinson, Franklin Lakes, NJ, USA) or visualized directly by fluorescent microscopy using the Olympus BX51 microscope equipped with a DP70 charge-coupled device camera (Olympus, Tokyo, Japan). As a positive control, *L. mexicana* wild type cells, treated by Hygromycin B, were used. Data were collected from three independent biological replicates and tested for the significance using two-tailed, paired Student *t*-test. *p* values ≤0.05 were considered statistically significant. 

### 4.5. Immunoblotting, Immunoprecipitation, and Mass Spectrometry Analysis

All used antibodies were from Sigma-Aldrich. Protein lysates (10 μg per lane) were separated in 10% SDS-PAGE, transferred and treated with polyclonal rabbit anti-HA (1:1,000) and monoclonal mouse anti-α-tubulin (1:2,000) primary antibodies, combined with HRP-labeled goat anti-rabbit IgG (1:80,000) and rabbit anti-mouse IgG (1:80,000) secondary antibodies, respectively. 

The immunoprecipitation of triple HA-tagged BnSP-7-ecDHFR was carried out with the *L. mexicana* protein lysates using Pierce Anti-HA magnetic beads (Thermo Fisher Scientific), following the manufacturer’s instructions. Elution of the sample was performed by heat denaturation in Laemmli sample buffer (Bio-Rad, Hercules, CA, USA) for 10 minutes. Samples were separated in 10% SDS-PAGE gels, stained with Coomassie Brilliant Blue G-250 (Thermo Fisher Scientific), cut out and sent for liquid chromatography–tandem mass spectrometry (LC–MS/MS) analysis (CEITEC, Brno, Czech Republic). 

### 4.6. Macrophage Infection

The *L. mexicana* wild type promastigotes were cultured in the M199 medium (Sigma-Aldrich) containing 10% FBS supplemented with 1% Basal Medium Eagle vitamins (Sigma-Aldrich), 2% sterile urine, and 250 µg/mL Amikacin (Bristol-Myers Squibb, New York, NY, USA), and 200 µg/mL of Hygromycin B (Carl Roth GmbH) for BnSP-7-ecDHFR-HA mutant cell lines. Infection of the J774 macrophages was performed as described previously [49,52]. In addition, infection was evaluated by counting Giemsa-stained slides as described previously [51]. All experiments were performed with two independent biological replicates and samples were analyzed in triplicate.

## Figures and Tables

**Table 1:** List of toxins/antimicrobial peptides fused with mut 2 ecDHFR-HA [22] and their toxic properties.

AMP/Toxin	Source	Mass (kDa)	Activity
Alpha-toxin	*Staphylococcus aureus*	35.9	plasma membrane permeabilization
Attacin-A	*Glossina morsitans morsitans*	21.7	plasma membrane permeabilization
BnSP-7	*Bothrops pauloensis*	13.7	plasma membrane permeabilization
Cathelicidin-5 (BMAP-28)	*Bos taurus*	17.6	plasma membrane permeabilization
Cecropin-A	*Hyalophora cecropia*	6.9	plasma membrane permeabilization
Exotoxin-A	*Pseudomonas aeruginosa*	69	elongation factor-2 inhibition

## Supplementary Materials

The following are available online at https://www.mdpi.com/2076-0817/9/2/79/s1, **Figure S1**: Growth curves of *Leishmania mexicana* encoding different toxins fused to ecDHFR destabilizing domain (mut 2 in [22]), with and without induction by stabilizing ligands, TMP and TMP-lac (20 µM). Red, wild type *L. mexicana*; violet, Attacin-A-ecDHFR-HA expressing *L. mexicana*; blue, Alpha-toxin-ecDHFR-HA expressing *L. mexicana*; neon green, BnSP-7-ecDHFR-HA expressing *L. mexicana*; brown, Cathelicidin-5-ecDHFR-HA expressing *L. mexicana*; green, Cecropin-A ecDHFR-HA expressing *L. mexicana*, **Figure S2:** Percentage of infected macrophages by the wild type and BnSP-7-ecDHFR-HA cell line non-treated (control) and treated with TMP-lac, **Table S1**: Growth kinetics of the wild type and BnSP-7-ecDHFR-HA-expressing *L. mexicana* after induction with 20 μM TMP/TMP-lac; standard deviations are indicated in the brackets, **Table S2**: List of primers used in this study.
