# Supplementary material for: Suicidal Leishmania
Source: Pathogens. 2020 Jan 25;9(2):79. doi: 10.3390/pathogens9020079 (PMC7168676; doi:10.3390/pathogens9020079)
Supplement: Supplementary file 1 [file pathogens-09-00079-s001.pdf]

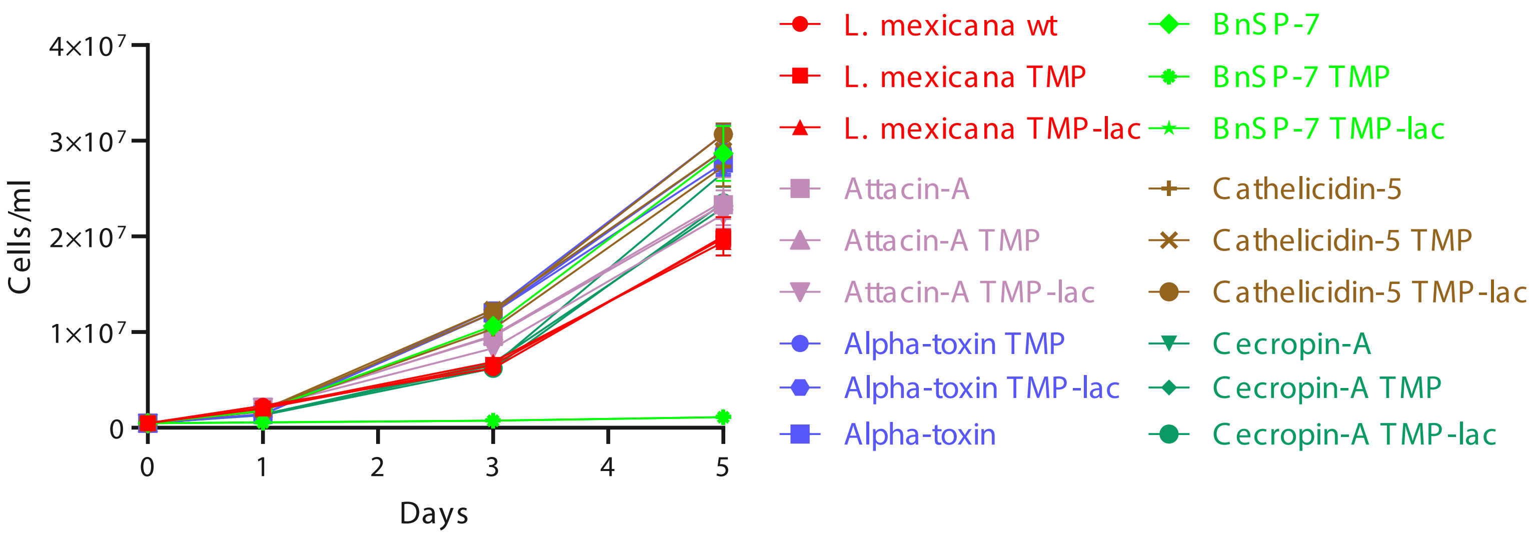

Figure S1: Growth curves of *Leishmania mexicana* encoding different toxins fused to ecDHFR destabilizing domain, with and without induction by stabilizing ligands, TMP and TMP-lac (20 μM).
Red, wild type *L. mexicana*; violet, Attacin-A-ecDHFR-HA expressing *L. mexicana*;
blue, Alpha-toxin-ecDHFR-HA expressing *L. mexicana*; neon green, BnSP-7-ecDHFR-HA expressing *L. mexicana*;
brown, Cathelicidin-5-ecDHFR-HA expressing *L. mexicana*; green, Cecropin-A ecDHFR-HA expressing *L. mexicana*

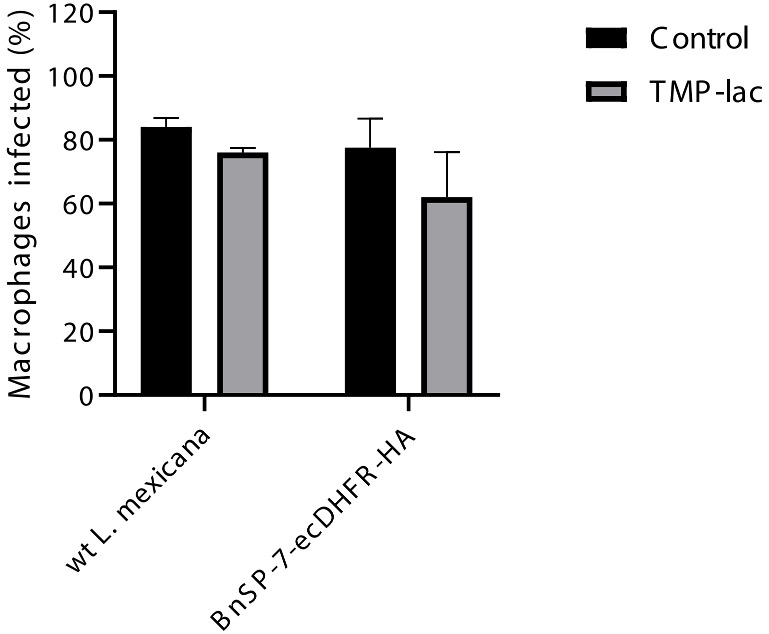

Figure S2: Percentage of infected macrophages by the wild type and BnSP-7-ecDHFR-HA cell line non-treated (control) and treated with TMP-lac

L. mexicana wt (cells/ml)			
Day	No ligand	TMP	TMP -lac
0	5,0E+05	5,0E+05	5,0E+05
1	2,3E+06 (2,6E+05)	2,0E+06 (5,8E+04)	2,1E+06 (1,2E+05)
3	6,2E+06 (3,4E+05)	6,6E+06 (5,3E+05)	6,9E+06 (3,1E+05)
5	2,0E+07 (2,0E+06)	2,0E+07 (9,2E+05)	1,9E+07 (2,0E+05)
BnSP -7-ecDHFR -HA (cells/ml)			
Day	No ligand	TMP	TMP -lac
0	5,0E+05	5,0E+05	5,0E+05
1	1,8E+06 (1,7E+05)	5,4E+05 (1,3E+05)	5,7E+05 (5,2E+04)
3	1,1E+07 (5,8E+05)	7,7E+05 (5,8E+04)	7,3E+05 (5,8E+04)
5	2,9E+07 (2,9E+06)	1,1E+06 (3,1E+05)	1,1E+06 (2,3E+05)

Table S1: Growth kinetics of the wild type and BnSP-7-ecDHFR-HA-expressing *L. mexicana* after induction with 20 µM TMP/TMP-lac; standard deviations are indicated in the brackets

Primer_name	Sequence
A1_alp-tox_BglII_F	gttctagcaagcagatctatgatgaaaatgaaaacacg
A2_alp-tox_R	ctaacgccgcaatcagactgatatttgtcatttcttcttttcccaatc
A3_alp-tox_fus_F	gcaagcagatctatgatgaaaatgaaaacacg
C1_cecr_BglII_F	gctgtgccttgccaccagatctatgaaatggaagttattcaagaagattg
C2_cecr_R	ctaacgccgcaatcagactgatacccttagcaatctgtgttgcc
C3_cecr_fus_F	ccttgccaccagatctatgaaatg
E1_exo_blunt_F	cttgccaccatgcacctgataccccattgg
E2_exo_R	ctaacgccgcaatcagactgatcttcaggtcctcgcgcg
E3_exo_fus_F	atgcacctgataccccattgg
D1_DDHA_F	atcagtctgattgcggcgttag
D2_DDHA_R	caaccgagaaggcgacgtg
D3_fus_R	aggaacaagaaaggaggaggagg
B1_Bnsp7_BglII_F	gccaccagatctatgagctttgaa
D4_DDHA3_R	atatagcggccgcctaggcgtagtcaggcacgtcatacggatatagcgcgtagtccggtacgtcatagggataaagagcgtaatctggaacatcgtatgg
SSU_F	catatgcttgtttcaaggac
HA_R	agcgtaatctggaacatcgtatggg

Table S2: List of primers used in this study
